# Diagnostic and Prognostic Significance of Exosomes and Their Components in Patients With Cancers

**DOI:** 10.1002/cam4.70569

**Published:** 2025-01-06

**Authors:** Zinnat Ara Moni, Zahid Hasan, Md. Shaheen Alam, Nitai Roy, Farhadul Islam

**Affiliations:** ^1^ Department of Biochemistry and Molecular Biology University of Rajshahi Rajshahi Bangladesh; ^2^ Department of Biochemistry and Molecular Biology Patuakhali Science and Technology University Patuakhali Bangladesh; ^3^ School of Medicine and Dentistry Griffith University Gold Coast Queensland Australia

**Keywords:** cancer progression, diagnosis markers, exosomes, survival

## Abstract

**Background:**

Cancer is the second leading cause of human mortality worldwide. Extracellular vesicles (EVs) from liquid biopsy samples are used in early cancer detection, characterization, and surveillance. Exosomes are a subset of EVs produced by all cells and present in all body fluids. They play an important role in the development of cancer because they are active transporters capable of carrying the contents of any type of cell. The objective of this review was to provide a brief overview of the clinical implication of exosomes or exosomal components in cancer diagnosis and prognosis.

**Methods:**

An extensive review of the current literature of exosomes and their components in cancer diagnosis and prognosis were carried out in the current study.

**Results:**

Tumor cells release exosomes that contribute to the formation of the pre‐metastatic microenvironment, angiogenesis, invasion, and treatment resistance. On the contrary, tumor cells release more exosomes than normal cells, and these tumor‐specific exosomes can carry the genomic and proteomic signature contents of the tumor cells, which can act as tools for the diagnosis and prognosis of patients with cancers.

**Conclusion:**

This information may help clinicians to improve the management of cancer patients in clinical settings in the future.

## Graphical abstract

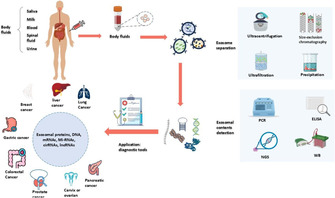

**Abbreviations**
AHSG: alpha‐2‐hs‐glycoprotein
AUC: area under the receiver operating characteristic curve
AUROC: area under the receiving operator curve
BBD: benign breast disease
BBT: benign breast tumor
BC: breast cancer
BIDs: benign intestinal diseases
BPH: benign prostatic hyperplasia
CA19‐9: carbohydrate antigen 19–9
CA72‐4: carbohydrate antigen 72–4
CC: cervical cancer
CEA: carcinoembryonic antigen
CHB: chronic hepatitis b
CI: confidence interval
CP: chronic pancreatitis
CRC: colorectal cancer
CRPC: castration‐resistant prostate cancer
csPCa: clinically significant prostate cancer
DFS: disease‐free survival
ECM1: extracellular matrix protein 1
EFS: event‐free survival
ELISA: enzyme‐linked immunosorbent assay
EOC: epithelial ovarian cancer
EVs: extracellular vesicles
FN: fibronectin
GC: gastric cancer
GKN1: Gastrokine 1
GLOBOCAN: global cancer observatory
HCC: hepatocellular carcinoma
HCs: healthy controls
HDs: healthy donor individuals
HR: hazard rate
IPMN: intraductal papillary mucinous neoplasm
LC‐ascites: liver cirrhosis‐associated ascites
LC–MS/MS: liquid chromatography mass spectroscopy
LMN: lymph node metastasis
LN: lymph node
LUAD: lung adenocarcinoma
mCRC: metastatic colorectal cancer
naPCa: non‐aggressive prostate cancer
NGS: next generation sequencing
NSCLC: non‐small cell lung cancer
OC: ovarian cancer
OPT: other pancreatic tumor
OS: overall survival
PB: pancreatic benign disease
PC 3R: docetaxel‐resistant prostate cancer (pc‐3) cells
PC: pancreatic cancer
PCa: prostate cancer
PCR: polymerase chain reaction
PDAC: pancreatic ductal adenocarcinoma
PFS: progression‐free survival
qRT‐PCR: quantitative reverse transcriptase‐polymerase chain reaction
ROC: receiver operating characteristic
SCC: squamous cell carcinoma
TEX: tumor‐derived exosomes
TME: tumor microenvironment
TNBC: triple‐negative breast cancer
TNM: tumor‐node‐metastasis
TTR: time to recurrence
WB: Western blotting

## 1 Introduction

One of the most potentially fatal multifactorial diseases is cancer, characterized by uncontrollable cell growth, invasion of nearby cells, and spread to other parts of the body, which is one of the top causes of death globally with a high rate of morbidity and mortality. According to the International Agency for Research on Cancer's GLOBOCAN 2022 estimates of cancer prevalence and mortality, there were 20.0 million new cases of cancer and about 9.7 million deaths due to cancer in the world in 2022 [1]. The three most hazardous malignancies in human lung, liver, and stomach cancers, with lung and breast cancers accounting for the majority of cancer‐related deaths in men and women, respectively. Prostate and thyroid cancer have the highest 5‐year survival rates (~100%), while esophageal, liver, and pancreatic cancer have the lowest rates (< 20%) [2]. Specific and effective biomarkers can improve early cancer detection, patient classification, and medication response prediction or prognosis, making them a potential disease management tool, which would lead to a better treatment outcome [3]. Liquid biopsy is gaining favor as a new cancer diagnosis method due to its unique characteristics such as non‐invasiveness, high sensitivity, and repeatability [4]. Liquid biopsies involve extracting tumor‐derived entities such as tumor extracellular vesicles (exosomes), which are prevalent in the bodily fluids of cancer patients, and then analyzing the genomic and proteomic data contained therein [5].

“Extracellular vesicles” (EVs) are particles that seem to be released from any type of cell, are bounded by a lipid bilayer, and are incapable of self‐replication (i.e., lack a functional nucleus). “Exosomes” are EVs (smaller than 200 nm) from internal cell compartments that are released through the multivesicular bodies (MVBs), a morphologically distinctive late endosome [6].

There is a strong correlation between the physiological and pathological states and the quantity, shape, composition, and content of exosomes that are discharged from the donor cells [7]. Exosomes play important roles in cancer pathophysiology because they contain proteins and nucleic acids (DNA/RNA) that can alter the microenvironment and accelerate the growth of cancer [8]. Tumor‐derived exosomes (TEX) possess an array of stimulatory and inhibitory factors that are involved in immune response regulation. These factors can impact the tumor microenvironment (TME), hence contributing to the development and advancement of cancer [9]. Through the transmission of heterogeneous payloads, exosomes can either activate or inhibit different signaling pathways in the recipient cells [10]. To assess the diagnosis, the expression of exosome biomarkers can compare between individuals with cancer and healthy individuals. To predict prognosis, biomarker extracted from tumor derived exosome can be analyzed by statistical tools to see the expression level and their correlation with chemotherapy resistance, overall survival (OS), disease‐free survival (DFS), recurrence, and metastasis [11]. Thus, it may be easier to manage cancer if exosomal contents are used as biomarkers for prognostic and diagnostic indicators [12].

To conduct basic research, exosomes must be isolated, detected, and quantified from non‐exosomal components such macrovesicles, apoptotic bodies, and other macromolecules in adequate amounts, purity, and size [13]. Exosome isolation and detection can be accomplished in a variety of ways, either conventional method or microfluidic‐based separation techniques. Conventional methods include size exclusion chromatography, precipitation, immunoaffinity, and ultracentrifugation (which is regarded as the gold standard). In contrast, microfluidic‐based separation techniques, such as nano‐biosensors, lab‐on‐chips, and nanoplatforms, are being researched recently to create next‐generation and extremely effective separation methods [14]. Although there are several ways to separate exosomes and new strategies are always being developed, each process has benefits and drawbacks [15]. Some of the disadvantages of employing these techniques include some being time‐consuming, the structure of exosomes may be disrupted by strong shear forces, the recovery rate may be intermediate, expensive equipment, possible loss of exosomes owing to membrane entrapment, contamination by other particles (e.g., lipoproteins), high reagent costs, and some are unsuitable for large‐scale exosome separation [16]. Exosomes generated from tumors are very heterogeneous. The identification of bulk exosomes could result in the omission of crucial data. Current approaches for analyzing exosome content cannot detect at the single exosome level, potentially leading to poor clinical performances. Therefore, additional studies on detection and isolation are required to lower the frequency of false‐negative cancer diagnosis outcomes [17]. The future development of novel liquid biopsy technology is expected to include a multidisciplinary process and ultimately have therapeutic application.

Thus, the purpose of this review is to present a thorough grasp of the clinical use of exosomal components, potentially contributing to a deeper comprehension of their involvement in the diagnosis and prognosis of cancers. Here, we performed a deep review in this field and searched the published papers in a number of academic databases, including Scopus, PubMed, Science Direct, Crossref, and Google Scholar. The literature that was found is retrieved and rigorously examined, and the findings are summarized herein.

### 1.1 Diagnostic Significance of Exosome in Cancers

**FIGURE 1:**
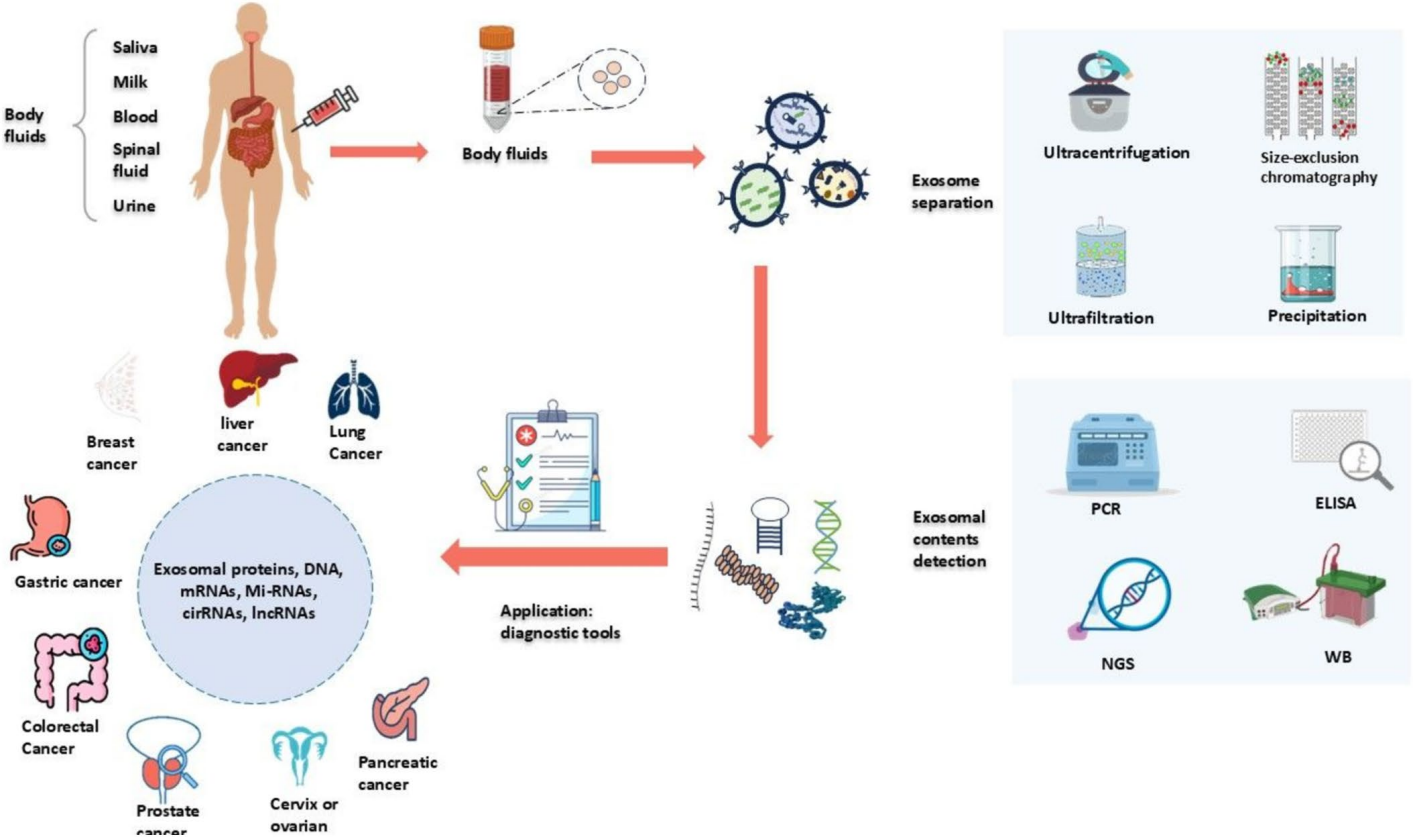
Exosomal contents in the diagnosis of various cancer. Early cancer identification can help determine the course of treatment or therapy for the specific cancer patient. Liquid biopsy provides an advantage over tissue biopsy for early cancer detection because it is noninvasive. Liquid biopsy isolates the tumor‐derived components such as circulating tumor cells and extracellular vesicles, especially exosomes. For the diagnosis of cancer, the body fluids (saliva, milk, blood, spinal fluid, and urine) are collected from the suspected cancer patients. From the body fluid, the exosomes are separated by ultracentrifugation, size exclusion chromatography, ultrafiltration, and precipitation processes. Then the presence of specific exosomal contents or biomarker or their expression level for specific cancer can be determined by polymerase chain reaction (PCR), next generation sequencing (NGS), Western blotting, and enzyme‐linked immunosorbent assay (ELISA). Different types of exosomal biomarker and different amounts of exosomal biomarkers indicate different types of cancer.

It is essential for tumor progression to undergo continuous carcinogenic reprogramming to develop malignant characteristics in the cells. Exosomes could act as a communication method for the transmission of onco‐signals, playing a dual role in tumorigenesis based on the originating cell. Exosomes not only serve as a means of communication but also have the potential to detect cancer in patients [18]. Exosomes or their components can assist in early cancer diagnosis and tracking of cancer development (Figure 1) [19]. Cancer cells secrete higher amounts of EVs than any other healthy cells [20]. This is why exosomes and their contents in particular have emerged as a potential source of information to analyze different types of cancers. The diagnostic implications of exosomes or their components in various cancers are illustrated in the following sections and summarized in Table 1.

**TABLE 1:** Diagnostic significance of exosomes or their components in patients with cancers.

Type of cancers	Exosomal components		Detection techniques	Clinical significance	Reference
Lung cancer or non‐small cell lung cancer (NSCLC)	Proteins	Alpha‐2‐HS‐glycoprotein (AHSG) and extracellular matrix protein 1 (ECM1), carcinooembryonic antigen	Ultracentrifugation	Increased significantly in the early‐stage NSCLC patients compared to the healthy control (HC).	[21]
		Olfactory receptors 9G9 and 2AT4. Homeobox proteins DBX1 and R‐spondin‐2	HPLC–MS	New potential diagnostic biomarkers for NSCLC patients.	[22]
		MUC1	LC–MS/MS	Plasma exosomal MUC1 level is higher in NSCLC patients than healthy individuals.	[23]
	Nucleic acids	Exosomal miR‐17‐5p	qRT‐PCR	Upregulated in NSCLC patients compared with the healthy controls (*p* < 0.001)	[24]
		Exosomal PLA2G10 mRNA	qRT‐PCR	Upregulation associated with higher stages, LMN and distant metastasis in patients with NSCLC.	[25]
		Exosomal miR‐23b‐3p	RT‐qPCR	Serum level higher in NSCLC patients than in patients with pneumonia (t = 10.332, *p* < 0.001); and healthy subjects	[26]
		Exosomal lncRNA DLX6‐AS1	RT‐qPCR	Upregulated in patients with NSCLC compared with in HCs.	[27]
		Exosomal lncRNA RP5‐977B1	qRT‐PCR	Exhibited higher levels in NSCLC than in the HCs.	[28]
		Exosomal lncRNA TBILA and AGAP2‐AS1	qRT‐PCR	Upregulated in NSCLC patients compared to HCs.	[29]
		Exosomal hsa_circ_0001492, 0001439, 0000896	qRT‐PCR	Upregulated in lung adenocarcinoma (LUAD) patients and associated with cancer progression.	[30]
		Exosomal hsa_circ_0069313	qRT‐PCR	Differentiate NSCLC and benign lung tumor and overexpression associated with stage III‐IV, LMN and distant metastasis of NSCLC.	[31]
		Exosomal circ_0048856	qRT‐PCR	Promote tumor growth and elevated in NSCLC serum with high diagnostic value.	[32]
		Exosomal miR‐1269a	qRT‐PCR	Diagnostic valve in patients with NSCLC with higher specificity and sensitivity.	[33]
		Exosomal miR‐1246	RT‐PCR	Increased in NSCLC patients and could discriminate NSCLC patients from HCs and patients with non‐malignant respiratory diseases.	[34]
		Exosomal LINC00917	qRT‐PCR	Highly expressed in NSCLC patients and associated with advanced stages patients.	[35]
Liver cancer or hepatocellular carcinoma (HCC)	Nucleic acids	Exosomal microRNA‐4661‐5p	qRT‐PCR	Significantly upregulated in HCC patients and has early‐stage diagnostic potential.	[36]
		Exosomal lncRNA (LINC00161)	qRT‐PCR	Upregulated in serum samples of HCC patients showed excellent stability and specificity (*p* < 0.001) in diagnosis.	[37]
		Exosomal lncRNAs ENSG00000258332.1 and LINC00635	TaqMan PCR	Overexpression associated with portal vein tumor emboli, LNM (lymph node metastasis), and TNM stage.	[38]
		Exosomal lncRNAs lnc‐FAM72D‐3 and lnc‐EPC1‐4	qPCR	Increased lnc‐FAM72D‐3 and decreased lnc‐EPC1‐4 levels are found in HCC when compared with controls.	[39]
Breast cancer (BC)	Proteins	Exosomal Del‐1	LC–MS/MS	Has potential for early‐stage BC diagnosis and distinguish BC from BBT and noncancerous diseases.	[40]
		Fibronectin	ELISA	Expression level significantly elevated (*p* < 0.0001) at all stages of BC.	[41]
		CD82	ELISA	Expression in BC tissue was significantly lower than that in healthy and benign breast disease (BBD) tissues.	[42]
	Nucleic acids	miR‐3662, miR‐146a, and miR‐1290	qRT‐PCR	Higher expression in serum exosomes in patients than HCs.	[43]
		miR‐363‐5p	Ultracentrifugation	Downregulated in exosomes from plasma of BC patients associated with LN metastasis.	[44]
		miR‐223‐3p	Ultracentrifugation, Microarray	Upregulated in patients with BC compared with the HCs.	[45]
		Exosomal lncRNA H19	qRT‐PCR	Upregulated in patients with BC compared to that in patients with BBD and HCs.	[46]
		Exosomal lncRNA XIST	RTqPCR	Serum level could discriminate patients with TNBC from HCs.	[47]
		Has_circ_0000615	qRT‐PCR	Higher expression associated with the advanced tumor stage (*p* = 0.010), lymph node metastasis (*p* = 0.001) and high grade of recurrence risk (*p* = 0.012).	[48]
Gastric cancer (GC)	Protein	TRIM3	LC–MS/MS	Downregulation in serum exosomes of GC patients than that in HCs.	[49]
	Nucleic acids	miR‐590‐5p	qRT‐PCR	Distinguish among healthy controls, early stage (I and II) group, and late stage (III) group.	[50]
		Exosomal miR‐181b‐5p	qRT‐PCR	Has diagnostic potential with better sensitivity than CEA.	[51]
		Exosomal miR‐1246	qRT‐PCR	Could differentiate early‐stage GC patients from HCs and patients with benign diseases.	[52]
		Exosomal miR‐423‐5p	RT‐PCR	Higher expression correlated with LNM.	[53]
		Exosomal lncRNA‐GC1	RT‐PCR	Significantly associated with GC from early to advanced stages with better sensitivity and specificity.	[54]
		Exosomal lncRNA MIAT	qRT‐PCR	Significantly higher in GC patients than in gastric adenoma patients and HC.	[55]
		Exosomal lncRNA CEBPA‐AS1	RT‐PCR	Can discriminate GC patients from HCs, with higher diagnostic accuracy.	[56]
		Exosomal lncRNA H19	qRT‐PCR	Upregulated in patients with GC both before and after surgery and preoperative expression level correlated with the TNM stage.	[57]
		Exosomal lncRNA lnc SLC2A12–10:1	qRT‐PCR	Has higher diagnostic accuracies than of CEA, CA 19–9, and CA72–4, correlated with tumor size, TNM stage, LNM, and degree of differentiation.	[58]
		Exosomal lncRNA lnc‐GNAQ‐6:1	qRT‐PCR	Downregulation has higher diagnostic accuracy than CEA, CA 19–9, and CA72–4.	[59]
		Exosomal lncRNA pcsk2‐2:1	qRT‐PCR	Downregulated correlated with tumor size (*p* = 0.0441), tumor stage (*p* = 0.0061), and venous invasion (*p* = 0.0367).	[60]
		Exosomal lncRNA HOTTIP	RT‐qPCR	Upregulation correlated with invasion depth (*p* = 0.0298) and TNM stage (*p* < 0.001).	[61]
		Exosomal miR‐92b‐3p, let‐7 g‐5p, miR‐146b‐5p, and miR‐9‐5p	NGS	Significantly associated with early‐stage GC (*p* < 0.05) and cancer progression.	[62]
		Exosomal circLPAR1	RT‐PCR	Lower plasma exosomal circLPAR1 was strongly linked to tumor size, differentiation degree, TNM staging, vascular invasion, LNM, and HER2 expression of GC patients (P < 0.05).	[63]
		Exosomal hsa_circ_000200	qRT‐PCR	Upregulation associated with invasion depth, TNM staging, and distal metastasis.	[64]
Colorectal cancer (CRC)	Proteins	Exosomal PLG and SERPINA1	ELISA	Has potential for early‐stage diagnosis of CRC and related to TNM staging.	[65]
		Exosomal CPNE3	RT‐PCR	Combined data from CEA and exosomal CPNE3 achieved 84.8% sensitivity and 81.2% specificity as a diagnostic tool.	[66]
	Nucleic Acids	Exosomal miRNA‐139‐3p	RT‐qPCR	Downregulation associated with metastatic colorectal cancer (mCRC) and submucosal patients.	[67]
		Exosomal circLPAR1	RT‐qPCR	Exhibited cancer specificity in CRC diagnosis.	[68]
		Exosomal hsa‐circ‐0004771	qRT‐PCR	Upregulation in serum of CRC patients compared to HCs and patients with benign intestinal diseases.	[69]
Prostate cancer (PCa)	Proteins	Exosomal PSMA	ELISA	Has diagnostic potential for patients with PCa with 95% sensitivity.	[70]
		Exosomal AMACR (UE‐A)	ELISA	Has the potential for diagnosis of early stages PCa patients.	[71]
		Exosomal ephrinA2	ELISA	Higher expression positively correlated with TNM staging.	[72]
	Nucleic acids	CD44v8–10 mRNA	RT‐digital PCR	Predict the therapy response in patients with PCa	[73]
		miR‐10a‐5p and miR‐29b‐3p	qRT‐PCR	Higher expressed in malignant compared to benign PCa patients.	[74]
		microRNA‐1246	qRT‐PCR	Downregulation associated with cancer progression.	[75]
		Hsa_circ_0044516	qRT‐PCR	Upregulated in the exosomes from PCa patients.	[76]
Cervical cancer (CC) or ovarian cancer (OC)	Proteins	CA125	Flow cytometry	Exosomal CA125 had improving the sensitivity of OC diagnosis.	[77]
	Nucleic acids	Exosomal miR‐125a‐5p	RT‐PCR	Expression levels in the patients with CC were lower than those in the HCs (*p* < 0.001).	[78]
		Exosomal miR‐651	qRT‐PCR	Has diagnostic potential with higher sensitivity and accuracy CC.	[79]
		Exosomal miR‐34a	qRT‐PCR	Differentiate early OC patient from advanced stages and also correlated LNM.	[80]
		Exosomal miR‐4732 5p	RT‐PCR	Upregulation in epithelial ovarian cancer (EOC), can distinguish EOC patients from healthy subjects (*p* < 0.0001).	[81]
		miR‐1307 and miR‐375	qRT‐PCR	Associated with tumor staging, LNM of OC.	[82]
Pancreatic cancer (PC)	Proteins	Exosomal protein c‐Met and PD‐L1	Flow Cytometry	Overexpression in pancreatic ductal adenocarcinoma (PDAC) in comparison to patients with benign disease.	[83]
		Exosomal ALIX	ELISA	Higher associated with the TNM stage and distant metastasis. In combination with serum CA199 can differentiate both early vs. late PC and PC vs. other pancreatic diseases.	[84]
		ALPPL2	Aptamer‐based ELISA (ALISA)	Expression level is high PC in EVs.	[85]
		Exosomal protein ZIP4	NanoLC‐MS/MS, ELISA	Upregulated in PC can compare between malignant PC patients and benign pancreatic disease patients and between biliary disease patients and HCs.	[86]
	Nucleic acids	Serum exosomal miR‐451a	RT‐PCR	Upregulation associated with clinical stage and distant metastasis in PC.	[87]
		Exo‐miR‐19b	RT‐PCR	Downregulated in patients with PC and discriminate other pancreatic diseases.	[88]
		ExmiR‐21 and ExmiR‐10b	Dynamic light scattering (DLS) technology	Upregulation can distinguish patients with early‐stage PC from controls and advanced stage PC.	[89]
		ExmiR‐191, ExmiR‐21 and ExmiR‐451a	qRT‐PCR	Upregulation has potential for early stages diagnostic markers of PC and IPMN.	[90]

### 1.2 Lung Cancer

Due to exosomes' role in various physiological and pathological processes, variations in the molecular composition and/or levels of exosomes from patients and healthy individuals could provide valuable information for the diagnosis and treatment of lung cancer [91]. Novel diagnostic biomarkers for early‐stage lung adenocarcinoma (LUAD) include circulating exosomal miR‐342‐5p and miR‐574‐5p, which showed significantly higher expression levels in carcinoma tissues compared to adjacent non‐cancerous tissues [92]. Identifying the increased levels of exosomal miR‐1246 and miR‐96 in NSCLC patients can be used as a diagnostic tool to differentiate between NSCLC patients and healthy individuals [93]. In addition, three exosomal circular RNAs, that is, circ_0047921, circ_0056285, and circ_0007761, have strong diagnostic validity for patients with NSCLC. When circ_0056285 and circ_0007761 are combined, they can distinguish NSCLC patients from tuberculosis controls; on the other hand, circ_0047921 can only differentiate NSCLC cases from chronic obstructive pulmonary disease controls [area under the curve (AUC) 0.820 versus 0.890] [94]. Through the activation of Ak strain transforming (AKT), the secretory protein Dickkopf‐1 (DKK1) binds to cytoskeleton‐associated membrane protein 4 (CKAP4), which in turn stimulates tumor growth. Compared to healthy controls (HCs), serum exosomal CKAP4 positivity was greater in lung cancer patients and serves as a lung cancer diagnostic biomarker [95]. Exosomal immunoglobulins generated from plasma that contain the IGHV4‐4 and IGLV 1–40 domains may offer novel diagnosis of NSCLC [96]. Patients with LUAD had significantly higher levels of hsa‐miR‐4454 and hsa‐miR‐619‐5p expression in their plasma exosomes with AUC values of 0.906 and 0.975, respectively. It may be possible to use these miRs as biomarkers for the early detection of lung cancer [97]. NSCLC patients had greater amounts of exosomal lncRNA RP5‐977B1 than did HCs. Exosomal RP5‐977B1 outperformed the traditional biomarkers CEA and CYFRA21‐1 in both the testing and multicentric validation cohorts, with an AUC value of 0.8899. Exosomal RP5‐977B1's diagnostic potential was also confirmed in early stages patients with NSCLC [28]. The tumor‐repressive function of exosome‐transported circ_0061407 and circ_0008103 is demonstrated, together with its diagnostic use in NSCLC. In addition, upregulation of these RNAs prevented NSCLC cells from proliferating, migrating/invading, and cloning in vitro, along with prevention of establishment of lung tumors in vivo [98]. The exosomal circRNAs hsa_circ_0001492, hsa_circ_0001439, and hsa_circ_0000896 are useful biomarkers for LUAD diagnosis (AUC value of 0.805) and the combination of these circRNAs demonstrated superior diagnostic sensitivity and specificity compared to a single marker [30]. Furthermore, serum‐derived exosomal piR‐hsa‐164,586 act as a novel biomarker for NSCLC early detection as its level elevated in cancer patients in comparison to that of blood samples from healthy people [99].

### 1.3 Liver Cancer

The lack of an effective early diagnosis method is the primary cause of liver cancer's lethality, making it the fourth leading cause of cancer‐related death [100]. Exosomes have been demonstrated in many studies to be useful as biomarkers for liver cancer detection [101]. Exosome marker proteins HSP70 and CD63 of tumor‐associated fibroblasts and exosomal miR‐92a‐3p act as potential biomarkers for HCC diagnosis [102]. Exosomal miR‐720 can be a valuable tool in identifying individuals with small HCC or elevated aminotransferase levels. It showed significant upregulation (fold change > 1.5) in HCC patients compared to non‐HCC individuals [103]. In liver cancer, exosomal miR‐92a‐3p and tumor fibroblast‐derived exosome marker proteins show good diagnostic value, suggesting that they could be used as novel diagnostic markers [102]. For example, exosomal hsa‐miR‐483‐5p act as a potential sensitive and specific biomarker for the diagnosis of HCC [104]. Potential biomarkers for the diagnosis of hepatocellular cancer include serum exosomal microRNA‐370‐3p and microRNA‐196a‐5p. Serum exosomes from patients with HCC showed increased expression of miR‐196a‐5p and lower expression of miR‐370‐3p. The TNM stage, tumor size, and tumor grade of HCC patients were correlated with serum exosomal miR‐370‐3p and miR‐196a‐5p [105].

Upon comparing the serum exo‐miR‐10b‐5p expression in the HCC group with the non‐tumorous group, a notable overexpression was observed [106]. Exosomal miR‐224 levels are significantly increased, indicating its potential as a diagnostic marker [107]. When examining the HCC group, exosomal microRNA‐4661‐5p showed a significant increase compared to the nontumor status. The result is highly precise compared to other serum markers for detecting HCC across all stages, with an area under the receiving operator curve (AUROC) of 0.917, and particularly in the early stages with an AUROC of 0.923. Therefore, a potential diagnostic indicator for early‐stage HCC is the serum panel based on exo‐miR‐4661‐5p [36]. In serum samples from HCC patients, exosomal lncRNA (LINC00161) was substantially enhanced with outstanding stability and specificity. The validated lncRNA signature's area under ROC curve was 0.794, indicating that circulating exosomal LINC00161 in serum could be a novel biomarker for HCC [37]. In comparison to controls, HCC has higher levels of exosomal lnc‐FAM72D‐3 and lower levels of exosomal lnc‐EPC1‐4. lnc‐EPC1‐4 and lnc‐FAM72D‐3 exhibit distinct functions that may contribute to the development of hepatocarcinogenesis and provide possible biomarkers for HCC identification [39].

### 1.4 Breast Cancer

Exosomal protein survivin was found in higher concentrations in BC patients than in healthy population [108]. BC patients showed a significant drop in serum exosomal miR‐940 expression. Increased expression levels indicate the lack of metastases in the lymph nodes [109]. Tumor‐derived exosomal hsa‐miR‐21‐5p, which is detected in peripheral blood, can efficiently discriminate between healthy people and cancer patients. It was highly upregulated in BC patients [110]. In BC patients, serum exosomal lncRNA DANCR levels are also elevated. The use of serum exosomal lncRNA DANCR in combination with A153 and CEA significantly improves diagnostic efficiency [111]. Also, exosomal miR‐92b‐5p offers a new management approach and serves as a non‐invasive biomarker for the diagnosis of BC. BC patients showed a substantial rise in exosomal miR‐92b‐5p and could distinguish patients from the control group (AUC of 0.787) [112]. Furthermore, serum exosomal miR‐200c may be a useful biomarker for BC diagnosis. The BC group had significantly reduced serum exosomal miR‐200c expression compared to the control and BBD groups. When paired with the traditional serum diagnostic markers aFP, CA125, and CA153, serum exosome miR‐200c can aid in increasing diagnostic efficacy [113]. Exosomal circulating miRNA‐373 and VEGF function as possible biomarkers for BC early detection. When it came to separating patients with early‐stage BC from HCs, ROC analysis showed that miRNA‐373 had a moderate discriminative power (specificity = 76.7%; sensitivity = 70.0%; AUC = 0.839), whereas exosomal VEGF had an excellent discriminative capacity (specificity = 85.0%; sensitivity = 90.0%; AUC = 0.944) [114]. In addition, two novel biomarkers for BC detection and tracking are exosomal miRNA‐21‐5p and miRNA‐10b‐5p. When comparing patients with BC grades I, II, and III to the control group, there was a significant increase in the expression of miRNA‐21–5p (*p* < 0.01), (*p* < 0.0001), and (p < 0.0001) respectively, as well as miRNA‐10b‐5p (*p* < 0.0001), (p < 0.0001), and (p < 0.0001), respectively [115]. TNBC can be detected early, thanks to new biomarkers for EDIL3 overexpressed exosomes, while EDIL3 is a powerful and very accurate diagnostic marker for BC. To diagnose TNBC, highly sensitive fluorescence detection of EDIL3 overexpressed exosomes is used [116]. Novel exosomal circEGFR promotes TFEB nuclear trafficking and modulates the miR‐224‐5p/ATG13/ULK1 feedback loop, which enables TNBC autophagy. Patients with BC had higher levels of circEGFR in plasma‐derived exosomes than did healthy individuals indicating as a possible therapeutic target and diagnostic biomarker for BC [117].

### 1.5 Gastric Cancer

Compared to the traditional GC inspection method, analyzing exosomal proteins provides faster results, easier sampling, lower discomfort and cost, and the potential to greatly improve GC diagnosis [118]. For example, serum exosomal HER2 level may be a useful biomarker to identify HER2 expression in cancer tissues in advanced GC and identifying possible patients who would benefit from anti‐HER2 treatment [119]. Also, salivary exosomal miR‐151‐3p may be a non‐invasive potential diagnostic for the diagnosis of GC [120]. In addition, serum gastrokine 1 (GKN1) protein is a highly specific and promising diagnostic biomarker for detecting both early‐stage and advanced GC. Healthy persons exhibited significantly greater serum GKN1 concentrations than patients with GC, and the best cutoff for serum GKN1 protein had a diagnosis accuracy of 0.9675 [121]. A study of 168 GC patients and 50 HCs found that GC patients in early and late stages had significantly lower levels of exosomal miR‐590‐5p expression (0.12‐fold, *p* < 0.05) than HCs [50]. Exosomal miR‐181b‐5p expression levels were determined using qRT‐PCR, which revealed that samples with benign liver cirrhosis‐associated ascites (LC‐ascites) had higher expression levels of exosomal miR‐181b‐5p than those with malignant GC associated ascites. It is a possible diagnostic biomarker for malignant ascites associated with GC, and exosomal miR‐181b‐5p and CEA profiling have been proven to produce the best diagnostic results [51]. MiR‐1246, which is found in circulating exosomes, could be used to detect GC early on. In vitro studies revealed that elevated serum miR‐1246 was derived from tumors and encapsulated into exosomes with the help of ELAVL1. Following that, it was discovered that GC patients with TNM stage I could be discriminated from HCs and patients with benign diseases by serum exosomal miR‐1246 expressions, which had AUCs of 0.843 and 0.811, respectively. Its serum expression could identify individuals with benign illnesses and HCs from GC patients at TNM stage I [52].

Exosomal lncRNA‐GC1, which is associated with GC, has a role in its development and can be used to assess GC early detection and progression. Individuals with GC had considerably higher levels of serum CEA and exosomal lncRNA‐GC1 than individuals with healthy donor (HDs). Furthermore, GC was strongly correlated with the levels of circulating exosomal lncRNA‐GC1 from early to advanced stages (HD vs. stage I, *t* = 20.98; *p* < 0.001; stage I vs. Stage II, t = 2.787; *p* = 0.006; Stage II vs. Stage III, t = 4.471; *p* < 0.001; Stage III vs. Stage IV, *t* = 1.023; *p* = 0.30) [54]. Patients with gastric adenoma and higher blood exosomal MIAT expression were more likely to develop GC. Exosomal lncRNA MIAT was significantly higher in GC patients than in gastric adenoma patients (*p* < 0.001) and HCs (*p* < 0.001) [55]. CEBPA‐AS1, an exosomal lncRNA, provides higher diagnostic accuracy than other traditional tumor biomarkers and can distinguish between GC patients and HCs. CEBPA‐AS1 had an AUC value of 0.824 when distinguishing between GC patients and GC [56]. GC patients had significantly higher amounts of exosomal lncRNA H19 both before and after surgery than HCs. The preoperative expression level and TNM stage showed a strong association. When evaluated separately or in combination, exosomal lncRNA H19 had a significantly higher AUC value of 0.849 than CEA, CA19‐9, and CA72‐4, and exosomal lncRNA H19 [57]. Compared to HCs, GC patients showed significantly greater levels of exosomal lncRNA lnc SLC2A12–10:1. The exosomal lnc‐SLC2A12–10:1 AUC value of 0.776 was significantly higher than the AUC values of 0.677, 0.660, and 0.633 for CEA, CA19‐9, and CA72‐4, respectively, the traditional cancer markers [58]. Compared to the HCs group, the GC group showed significantly lower levels of exosomal lncRNA (lnc‐GNAQ‐6:1), but had a higher diagnostic accuracy than CEA, CA 19–9, and CA 72–4 [59]. According to qRT‐PCR findings, the expression level of Lnc RNA PCSK2‐2:1 was significantly lower in the serum exosomes of GC patients compared to HCs (*p* = 0.006), with diagnostic sensitivity and specificity of 84% and 86.5%, respectively [60].

The combination of four serum exosomal miRNAs (miR‐92b‐3p, let‐7 g‐5p, miR‐146b‐5p, and miR‐9‐5p) is a novel diagnostic biomarker for early‐stage GC. Higher levels of expression were found to be substantially linked with early‐stage GC (*p* < 0.05). In early‐stage GC, serum levels of exosomal miR‐92b‐3p were statistically correlated with tumor invasion depth (*p* = 0.0089), let‐7 g‐5p and miR 146b‐5p were significantly correlated with nerve infiltration (*p* = 0.0234 and 0.0126, respectively), and exosomal miR‐92b‐3p was significantly associated with poor cohesiveness (*p* = 0.0021) [62]. GC patients exhibited lower plasma exosomal circLPAR1 levels than chronic gastritis patients and HCs (*p* < 0.05), but post‐surgical GC patients had greater levels (*p* < 0.05). Low plasma exosomal circLPAR1 was found to be significantly linked with tumor size, differentiation level, TNM staging, vascular invasion, lymphatic metastasis, and HER2 expression in GC patients (*p* < 0.05) [63]. Hsa_circ_000200 levels were higher in the serum and exosomes of GC patients. The results show that hsa_circ_000200 has a greater diagnostic efficiency for serum exosomes, with AUC values of 0.7092 [64].

### 1.6 Colorectal Cancer

Exosomes can accelerate the development of colorectal cancer (CRC) by promoting the growth of tumor cells, decreasing apoptosis by modification of key regulatory genes, or modulating several signaling pathways. Therefore, exosomes derived from CRC could be an important biomarkers for CRC diagnosis [122]. Patients with CRC showed significantly higher levels of fibrinogen (PLG) and serpin peptidase inhibitor clade A member 1 (SERPINA1) compared to the HCs. The ROC curve analysis outperformed both CEA and CA19‐9 in diagnosing CRC, especially at the early stages of CRC [65]. For instance, exosomal elevated expression of CD147 may be a biomarker for CRC diagnosis and prognosis. CEA, CA19‐9, and CD147‐specific exosomes (exo‐CD147) were used to characterize and evaluate ROC curves between HDs and patients with CRC patients. The exo‐CD147, CEA, and CA19‐9 AUC were, respectively, 0.827 (95%CI: 0.764–0.891), 0.630 (95%CI: 0.536–0.724), and 0.659 (95%CI: 0.559–0.759) [123]. In addition, exosomal protein CPNE3 in circulation serves as a biomarker for the diagnosis of patients with CRC. Compared to HCs, exosomal CPNE3 levels in CRC patients were significantly elevated. Furthermore, in comparison to stage I–II patients, stage III–IV patients exhibited elevated levels of CPNE3 expression in their plasma exosomes [66].

CRC patients showed a substantial upregulation of exosomal miR‐1470 in comparison to healthy individuals. With an AUC of 0.74 (95% CI: 0.6876–0.7920), exosomal miR‐1470 showed promise as a diagnostic biomarker according to the ROC curve analysis [124]. Also, exosomal miR‐205‐5p in serum serves as a CRC diagnostic biomarker. The serum of CRC patients had a lower level of exosomal miR‐205‐5p than that of patients with benign disease (*p* < 0.0001) and HCs (p < 0.0001). Additionally, compared to the control groups, early‐stage CRC patients had significantly reduced levels of exosomal miR‐205‐5p expression (*p* < 0.001 and p < 0.0001) [125]. CircGAPVD1 generated from plasma exosomes serve as a possible CRC diagnostic marker. According to ROC analysis, upregulation of circ GAPVD1 in plasma exosomes may be useful for CRC diagnosis and is shown to have a sensitivity of 75.64 and a specificity of 71.79% in the diagnosis with AUROC = 0.7662 [126]. Plasma exosomal miRNA‐139‐3p is a blood‐based biomarker for early detection and metastatic tracking of CRC. Its expression levels were significantly lower in CRC patients than in HCs [67]. The expression of CircLPAR1 in plasma exosomes was markedly reduced throughout the development of CRC. Furthermore, CircLPAR1 was enclosed within exosomes, exhibiting exceptional stability and detectability. The exosomal circLPAR1 inhibits BRD4 through the METTL3–eIF3h interaction, leading to a reduction in the translation of the oncogene BRD4. This process contributes to the identification and development of CRC [68]. qRT‐PCR analysis indicated that the serum of CRC patients had elevated expression of exosomal hsa circum‐0004771 compared to both HCs and patients with benign intestinal diseases (BIDs). The exosomal hsa‐circ‐0004771, which is present in the bloodstream, was discovered to exhibit AUROC and AUCs of 0.59, 0.86, and 0.88 for distinguishing CRC patients from HCs, BIDs, and stage I/II CRC patients, respectively [69].

### 1.7 Prostate Cancer

PCa is the second leading cause of cancer‐related deaths among males in developed countries. Conventional treatments for PCa are not successful for patients with advanced or metastatic cancer [127]. The plasma of PCa patients has shown higher levels of carbonic anhydrase IX (CA‐IX) expression, suggesting that this biomarker could be valuable for detecting malignancy [128]. Exosomes contain several components such as mRNAs, microRNAs, non‐encoded RNA, proteins, and lipids. These components have been utilized in the evaluation of tumors and as indicators of tumors for early diagnosis and detection of PCa [129]. The PCa groups demonstrated significantly elevated levels of exosomal protein PSMA compared to the BPH and BPH + non‐aggressive prostate cancer groups (*p* < 0.001). This indicates that exosomal protein PSMA has a high diagnostic accuracy for diagnosing PCa [70].

The expression of exosomal protein AMACR in PCa was significantly greater than in BPH and non‐aggressive cases (*p* < 0.001). A multi‐center cohort of patients further confirmed the effectiveness of urine exosomal AMACR in identifying PCa, with an AUC of 0.800. This test can be utilized for the early identification of PCa [71]. Docetaxel is the primary treatment for castration‐resistant prostate cancer (CRPC), although a significant number of patients acquire resistance to this medication. CD44v8–10 has been found to be potentially linked to docetaxel‐resistant prostate cancer. Furthermore, the presence of serum exosomal CD44v8–10 mRNA could potentially be used as a diagnostic indicator for this specific form of cancer. A notable disparity (*p* = 0.032) in serum exosomal CD44v8–10 mRNA was observed between docetaxel‐resistant individuals and control males [73]. Exosomal miR‐10a‐5p and miR‐29b‐3p have been identified as potential indicators for detecting PCa. These markers were identified by low input NGS research and were further confirmed through retesting. The expression levels of exosomal miR‐10a‐5p and miR‐29b‐3p were significantly higher in malignant EVs from PCa patients compared to benign EVs [74]. This finding can potentially improve the accuracy of diagnostic predictions for PCa. The levels of exosomal Hsa_circ_0044516 were shown to be significantly greater in both PCa patient exosomes and cell lines. Additionally, the downregulation of circ_0044516 hindered the growth of PCa cells [76].

### 1.8 Cervix Or Ovarian Cancer

Ovarian cancer is one of the three most prevalent malignant tumors of the female reproductive system that risk women's health. CA125, an exosomal protein, could be a useful diagnostic for OC. Exosomes contain larger quantities of CA125 than serum, increasing the sensitivity of OC diagnosis [77]. There have been studies relating exosomal microRNAs (miRs/miRNAs) to CC. Circulating exosomal miR‐125a‐5p is a new biomarker for CC. Exosomal miR‐125a‐5p expression was significantly lower in CC patients compared to HCs (*p* < 0.001). According to ROC curve analysis, plasma exosomal miR‐125a‐5p is a promising marker for distinguishing CC from non‐CC, with an AUROC of 0.7129 [78].

Cancer patients reported lower levels of circulating exosomal miR‐651 than healthy individuals. It had an AUC of 0:9050 and showed high sensitivity and accuracy in the diagnosis of CC [79]. Individuals in the early stage of OC exhibited significantly higher blood exosomal miR‐34a levels than those in the advanced stage of the disease. Serum exosomal miR‐34a may be an excellent biomarker for OC diagnosis [80]. Exosomal miR‐4732‐5p collected from plasma may be a useful biomarker for detecting epithelial OC. Exosomal miR‐4732‐5p from plasma had an AUROC curve of 0.889, indicating a sensitivity of 85.7% and specificity of 82.4% in distinguishing early OC patients from healthy people (*p* < 0.0001) [81]. The levels of miR‐1307 and miR‐375 in OC serum exosomes have been found to remain steady. Furthermore, OC serum exosomes contained significantly higher amounts of miR‐1307 and miR‐375 than the groups with benign OC and those in good health. In addition to CA‐125 and HE4, overexpressed miRNAs revealed independent diagnostic capabilities and increased the diagnostic precision of conventional biomarkers. MiR‐375 was linked to OC lymph node metastasis, whereas miR‐1307 was linked to tumor stage [82]. For the diagnosis of CC, plasma exosomal circSLC26A4 can be utilized. Compared to healthy women and high‐grade squamous intraepithelial lesion patients, CC patients had significantly greater plasma exosomal circSLC26A4 levels (*p* < 0.05). Furthermore, FIGO stage and lymph node metastases were positively correlated with increased plasma exosomal circSLC26A4 expression (all *p* < 0.05) [130].

### 1.9 Pancreatic Cancer

Typically, pancreatic cancer is detected at an advanced stage due to its highly metastatic nature. To potentially find a cure for PC, it is crucial to identify and create biomarkers for early detection [131]. Investigating the diagnostic potential of exosomal proteins c‐Met and PD‐L1 in PC. When examining patients with benign disease versus those with pancreatic ductal adenocarcinoma (PDAC), it was found that the latter group exhibited elevated levels of exosomal PD‐L1 and c‐Met. Based on a c‐Met diagnostic test, a sensitivity of 70% and a specificity of 85% were obtained [83]. When examining patients with PC compared to those with other pancreatic conditions or healthy individuals, the expression of the exosomal protein ALIX was found to be elevated in the former group. It showed a strong correlation with the TNM stage and distant metastases [84]. ALPPL2, an exosomal protein, shows significant expression in extracellular vesicles from PC and could serve as a potential diagnostic biomarker [85].

Immune lipoplex nanoparticle biochip assay showed GPC1 membrane protein expression in the microvesicle‐rich EV subpopulation, especially the tumor‐associated microvesicles, and Glypican1 (GPC1) mRNA expression in the exosomes‐rich (Exo)EV subpopulation served as a viable biomarker for PDAC [132]. The in situ‐proven ExmiR 4516 may be a promising tool for liquid assays that monitor the clinical progression of PDAC and detect it at a relatively early stage and the sensitivity, specificity, and accuracy of ExmiR‐4516 were 97.5%, 34.3%, and 68%, respectively [133]. When examining serum‐derived exosomes from PC patients versus those from healthy individuals, a significant increase in miR‐451a expression was observed. Identifying patients with PC is highly effective using serum exosomal miR‐451a for diagnosis. Moreover, exosomal miR‐451a showed a significant association with the clinical stage and distant metastases of PC [87]. One promising diagnostic marker for a certain type of cancer is plasma‐derived Exo‐miR‐19b. When differentiating between patients with PC and healthy volunteers, the diagnostic effectiveness of plasma‐derived Exo‐miR‐19b adjusted using miR‐1228 surpasses that of serum CA19‐9 [88]. Individuals with PC showed notably elevated levels of exmiR‐21 and exmiR‐10b in comparison to the control group. When combined with exmiR‐10b, the diagnostic significance of exmiR‐21 was enhanced (AUC = 0.791). ExmiR‐21 demonstrated the capability to distinguish patients with early‐stage PC from both controls and advanced stage PC (early‐stage vs. advanced stage; early‐stage vs. healthy) [89]. Two exosomal circular RNAs, namely, hsa_circ_0001666 and hsa_circ_0006220, can be used as markers for detecting PC. Hsa_circ_0006220 and hsa_circ_0001666 exhibited a substantial increase in expression levels inside exosomes present in the plasma of individuals diagnosed with PC, in comparison to individuals without the disease. The AUC values for hsa_circ_0006220, hsa_circ_0001666, and the combined diagnosis were 0.7817, 0.8062, and 0.884, respectively. These results indicate that exosomal circular RNAs have the potential to serve as biomarkers for the early detection of PC [134].

Altogether, current information from preclinical and clinical studies implied the diagnostic significance of exosomes, especially their contents in various types of cancers. However, the lack of effective and well‐accepted methods of detection and characterization and standardized protocols for their clinical utilization limit their application in clinical settings. Therefore, development of standard operating procedures and validation of their diagnostic roles with multicenter large‐cohort analysis for various cancer types need to be established before their translation applications.

### 1.10 Prognostic Significance of Exosome in Cancers

**FIGURE 2:**
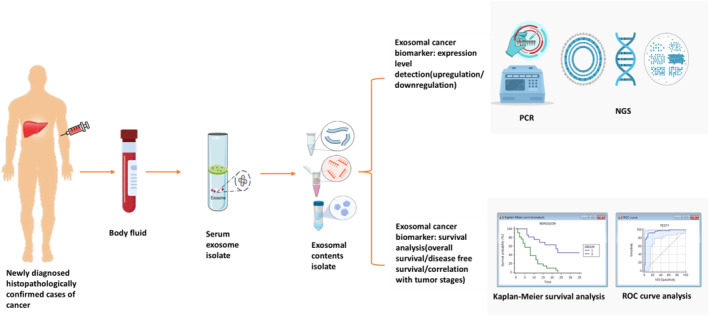
Exosomal contents as prognostic biomarker for cancer patients. After confirming that the individual has cancer, a clinician can use the exosomal biomarker to evaluate the disease progression. For this, body fluid can be collected from newly diagnosed histopathologically confirmed cases of cancer patients; from there, the exosomes need to be collected, and finally, the exosomal contents such as proteins, DNAs and RNAs can be separated (mRNAs, miRNAs, and lncRNAs). By using the real time PCR and next generation sequencing (NGS), the expression level (upregulation/downregulation) of the exosomal contents can be determined. Further analysis of these contents by statistical survival analysis including Kaplan–Meier survival analysis, receiver operating characteristic curve (ROC curve) for estimating the OS, disease‐free survival, and also grading tumor stages.

A prognosis is a forecast of how a disease will progress that most clinicians find challenging to explain. Since cancer is a heterogeneous disease, its prognosis takes a lot of research [135]. Additionally, a great deal of research interest leads to the identification of exosomal biomarkers for prognostic significance (Figure 2).

### 1.11 Lung Cancer

Exosomes play a crucial role in determining lung cancer prognosis, and their molecular components can be targeted to disrupt cellular signaling. They also have the potential to serve as biomarkers for predicting cancer outcomes [91]. The expression level of exosomal components showed a strong correlation with both OS and DFS in lung cancer patients, suggesting a link between exosomes and a negative prognosis for the disease [136]. Studying exosomal proteins reveals their significance as essential exosome contents that play a vital role in facilitating cell communication and influencing various aspects of tumor development, including angiogenesis, epithelial–mesenchymal transition, metastasis, immune responses related to tumors, and reshaping the microenvironment. Thus, characterization of exosomal proteins can provide insights into exosome production, targeting, and function. These could potentially be valuable as prognostic indicators for cancer [12]. In patients with NSCLC, out of the 1220 exosomal proteins examined, two distinct panels of biomarkers were identified. The initial panel, including FGB, FGG, and VWF, displayed potential for early NSCLC detection and exhibited a correlation with the survival period of NSCLC patients. CFHR5, C9, and MBL2, the second panel of biomarkers, displayed potential as biomarkers for assessing NSCLC metastasis; specifically, CFHR5 alone was significantly associated with the OS of NSCLC patients [137]. Also, BTG‐1 protein derived from plasma exosomes may serve as a valuable biomarker for predicting the prognosis of NSCLC patients. According to the findings of a multivariate Cox regression analysis, individuals who exhibited lower levels of BTG‐1 in their plasma exosomes experienced shorter DFS and OS in comparison to those with higher levels of BTG‐1. Based on the findings, it was observed that BTG‐1 produced by plasma exosomes demonstrated a sensitivity of 91.0% and a specificity of 82.3% for 3‐year DFS, as well as a sensitivity of 81.7% and a specificity of 93.0% for 3‐year OS [138]. In addition, serum exo‐eIF4E derived from tumors can provide valuable insights into predicting the prognosis of NSCLC. Exo‐eIF4E has been identified as a significant predictor for reduced OS and PFS based on the combined multivariate and univariate analysis. Patients with NSCLC higher levels of exo‐eIF4E demonstrate a higher likelihood of experiencing reduced OS and poorer PFS [139].

NSCLC patients with a significant increase in miRNA‐214 expression, greater than 17‐fold, may indicate a less favorable prognosis. The research discovered a link between higher levels of miRNA‐214 in the blood and the advancement of the disease. It suggested that measuring miRNA‐214 in the blood could potentially be used to predict patient outcomes and identify those with a lower chance of survival in cases of NSCLC [140]. Patients diagnosed with NSCLC exhibited a notable increase in the levels of serum exosomal miR‐378 expression. There was a clear connection observed between elevated levels of serum exosomal miR‐378 expression and the presence of positive LNM, as well as the progression to advanced TNM stage. Based on survival analyses, it was observed that patients with higher plasma levels of exosomal miR‐378 had a lower OS rate. Also, the expression of serum exosomal miR‐378 had a significant correlation with OS, as determined through multivariate analysis [141]. Serum exosomal miR‐1246 could potentially serve as a valuable biomarker for predicting the prognosis of NSCLC. There was a strong correlation between the levels of serum exosomal miR‐1246 and both the TNM stage and LNM. A higher serum exosomal miR‐1246 group experienced lower DFS and OS rates [34]. A positive correlation was discovered between low clinical variables and decreased levels of exosomal miR‐382 in circulation. There is a clear relationship between the OS of NSCLC cases and the levels of serum exosomal miR‐382. A lower exosomal miR‐382 had a lower OS [142]. In comparison to the HCs, NSCLC exhibited higher levels of exosomal lncRNA RP5‐977B1. In NSCLC, a higher expression of exosomal RP5‐977B1 was found to be significantly correlated with a poorer prognosis [28]. There was a notable disparity in the PFS observed among LUAD patients who exhibited a high level of exosomal miR‐1290 compared to those with a low level. In Cox proportional hazards model, it has been found that exosomal miR‐1290 could potentially serve as an independent prognostic factor for LUAD [143]. The serum exosomal lncRNA SNHG15 has been confirmed as a reliable predictor for OS in various studies, both univariate and multivariate. Patients diagnosed with NSCLC and certain characteristics, such as higher serum exosomal lncRNA SNHG15 expression, poor differentiation, positive LNM, or advanced TNM stage, experienced shorter OS rates. The statistical analysis revealed significant correlations between these factors and OS [144].

### 1.12 Liver Cancer

Hepatocellular carcinoma is a highly prevalent form of cancer that is seen worldwide. Due to the intricate nature of HCC onset and the lack of specific early‐stage indicators, the diagnosis and treatment of HCC in its early stages are still insufficient, leading to an unfavorable prognosis [145]. A study has been conducted on the impact of FTCD (a potential exosome‐related biomarker) on the prognosis of HCC and its role in promoting M1 macrophage polarization. FTCD showed statistical significance in the multivariate Cox regression model, suggesting its potential as a biomarker for prognostic assessments of HCC [146]. Patients with HCC who have undergone surgical treatment may have their prognosis determined by the presence of TP53 mutations in exoDNA. Studies have shown that individuals with a higher frequency of mutations experienced a shorter median time until recurrence of the disease, with a range of 68–202 days. On the other hand, patients with a lower frequency of mutations had a longer median time until recurrence, ranging from 368 to 576 days. In multivariable Cox regression analysis, it was discovered that a high frequency of mutations in the TP53 gene observed in exoDNA (MD/TD ≥ 67%) was strongly associated with a poor prognosis [147].

In HCC, miR‐122 and miR148a have been identified as powerful predictive biomarkers. It has been observed that a low expression level of these exosomal miRNAs is associated with a lower survival rate. This finding was obtained using KM‐Plotter [148]. Also, serum exosomal microRNA‐370‐3p and microRNA‐196a‐5p has prognostic significance in HCC patients. The expression of miR‐370‐3p shows a correlation with tumor size, TNM stage, and tumor grade. Additionally, patients with low expression had a significantly shorter 3‐year OS compared to those with high expression, indicating that high expression is associated with a lower risk of death. In contrast, there is a correlation between the expression of miR‐196a‐5p and various tumor characteristics such as size, TNM stage, and grade. Additionally, patients with a low level of miR‐196a‐5p had a significantly longer 3‐year OS compared to those with a high level. Thus, individuals with a higher expression of miR‐196a‐5p in serum exosomes had a greater risk of death compared to those with lower expression [105]. There was a strong correlation between shorter survival, advanced TNM stage, positive LNM, and positive vein invasion with the downregulation of serum exosomal miR‐320a. The validation of serum exosomal miR‐320a as an independent predictive marker for HCC has been confirmed. In Kaplan–Meier survival analysis, it was observed that patients in the group with low serum exosomal miR‐320a expression had a lower OS compared to those in the high expression group [149]. Tumors at an advanced stage, the presence of metastases in lymph nodes, and poorly differentiated cancers were found to be associated with decreased levels of miR‐320d in the serum. When the blood exosomal miR‐320d levels were reduced, patients with HCC experienced a decrease in both OS and DFS. Serum exosomal miR‐320d could potentially provide valuable insights into the prognosis of HCC, offering a non‐invasive approach for prediction [150]. Survival analysis showed that patients with lower serum exosomal miR‐34a expression experienced a more unfavorable overall prognosis compared to patients with higher levels of expression. There is a clear and independent relationship between serum exosomal miR‐34a and OS, as shown by the results of both univariate and multivariate Cox regression analysis [151]. Patients who exhibited higher levels of circulating exosomal miRNA‐21 (≥ 0.09) and lncRNA‐ATB (≥ 0.0016) experienced notably worse OS and PFS (log‐rank test: *p* < 0.05). In addition to larger tumor size and elevated C‐reactive protein, a comprehensive analysis using the Cox regression test demonstrated that increased levels of miRNA‐21 and lncRNA‐ATB were significant indicators of both mortality and disease progression [152]. Combining serum exosomal ENSG00000258332.1, LINC00635, and AFP could potentially serve as a valuable test for assessing the prognosis of HCC. Survival rates, the spread of cancer to lymph nodes, the presence of tumor emboli in the portal vein, and the stage of the cancer were all found to be correlated with elevated levels of a specific gene in HCC (Table 1).

### 1.13 Breast Cancer

Currently, breast cancer was considered the primary cause of death among women because it was the most frequently detected type of cancer. Based on data from the SEER database (Female Breast Cancer—Cancer Stat Facts; https://seer.cancer.gov/statfacts/html/breast.html) approximately 91.2% (2014–2020) of patients diagnosed with breast cancer can anticipate a 5‐year survival rate. According to reports, there were approximately 3,972,256 women in the United States who were living with female breast cancer in 2021. In 2024, there are projected to be approximately 310,720 new cases (15.5%) and an estimated 42,250 deaths (6.9%). The percentage of cancer deaths from female BC in 2023 was 7.1%. Thanks to advancements in diagnosis and treatment, the mortality rate of BC has shown a decline over the years. However, given its diverse nature, it is crucial to assess prognostic indicators in order to determine the most effective treatment approach [153].

Exosomal mRNAs (e.g., NANOG, NEUROD1, HTR7, KISS1R, and HOXC) have been identified as potential prognostic biomarkers for BC patients. They have shown significant associations with OS (*p* = 0.016) and DFS (*p* = 0.021) [154]. It was found that individuals with high exosomal HOTAIR expression had a lower OS rate and DFS rate compared to those with low exosomal HOTAIR expression in BC. A strong correlation was found between elevated levels of serum exosomal HOTAIR and poor response to neoadjuvant chemotherapy and tamoxifen therapy [155]. It has been observed that BC patients with a lower level of serum exosomal miR‐148a expression had a shorter 5‐year DFS. The multivariate analysis revealed that serum exosomal miR‐148a, LNM, and TNM stage did not emerge as significant predictors of BC [156]. Significant correlations were found between elevated levels of miR‐1246 and miR‐155 and unfavorable event‐free survival (EFS) in patients at the early stages of their illness, as well as poor PFS in patients with metastatic disease. These findings were determined through rigorous predictive variables and multivariate analysis. In Kaplan–Meier survival analysis, it was observed that patients with metastatic or early‐stage disease and higher expression of miR‐155 and miR‐1246 had a more unfavorable prognosis in terms of PFS and EFS compared to patients with lower expression of both miRNAs [157]. Patients with elevated blood exosomal lncRNA DANCR expression showed a significantly reduced overall 5‐year survival period in cases of BC. Through multivariate analysis, it was determined that the presence of serum exosomal lncRNA DANCR is an independent risk factor for BC. The Kaplan–Meier curve illustrates the OS in relation to the expression of serum exosomal lncRNA DANCR. It was observed that patients with high expression of serum exosomal lncRNA DANCR had a notably shorter survival time (*p* = 0.0132) compared to BC patients with low expression [111]. The presence of exosomal lncRNA SUMO1P3 in serum could potentially serve as a reliable and robust predictive biomarker for TNBC. Patients in the high serum exosomal lncRNA SUMO1P3 group exhibited a decreased OS when compared to those in the low serum exosomal lncRNA SUMO1P3 group [158].

### 1.14 Gastric Cancer

Emerging studies indicate that exosomes play a vital role in the onset and progression of stomach cancer. For example, serum exosomal miR‐590‐5p as a potential prognostic biomarker for GC. A strong correlation was observed between the decrease in the explicit level of exosomal miR‐590‐5p and the decline in the OS rate [50]. Exosomal miRNA‐23b shows potential as a non‐invasive prognostic biomarker for gauging the prognosis and recurrence of GC in patients at all stages of the disease. In stages I, II, III, and IV, the OS rates of patients with low levels of miR‐23b were significantly lower compared to those with high levels of miR‐23b. In stages I, II, and III, patients with low exosomal miR‐23b had significantly lower DFS rates compared to patients with high miR‐23b [159]. A potential novel prognostic biomarker test for GC is exosomal lncRNA HOTTIP. There was a strong correlation between higher levels of exosomal HOTTIP and lower OS. In addition, it was discovered that the overexpression of exosomal HOTTIP is an independent predictive factor in patients with GC, as determined by both univariate and multivariate COX analysis (*p* = 0.027) [61]. When analyzing the high exosomal PD‐L1 group and the low exosomal PD‐L1 group, it was observed that OS was significantly lower (*p* = 0.004). Exosomal PD‐L1 is a valuable indicator of the immunological state and can provide insights into the prognosis of patients with GC [160]. After surgery, the serum exosomal lncRNA H19 levels in patients with GC were significantly lower compared to their pre‐surgery levels. The strong correlation between TNM stage and preoperative lncRNA H19 levels suggests that circulating exosomal lncRNA H19 could serve as a valuable biomarker for GC prognosis [57]. A new prognostic biomarker for GC has been discovered in the form of serum exosomal lncRNA MIAT. A significant link was discovered between the overexpression of serum exosomal MIAT and unfavorable clinical characteristics, along with a shorter survival period [55]. The connection between OS and DFS and circulating exosomal lncRNA‐GC1 was found to be significant (*p* < 0.05). By employing multivariate analysis, it was discovered that circulating exosomal lncRNA‐GC1 and stage independently predicted the outcome of GC (*p* < 0.05). These findings suggest that the presence of exosomal lncRNA‐GC1 in the bloodstream can serve as a reliable indicator for prognosis of patients with GC [161].

### 1.15 CRC

CRC is a dangerous malignant tumor due to its comparatively high incidence and fatality rates. Exosomes or its components have the potential to greatly increase CRC therapy options, increase prognostic precision, and improve diagnosis accuracy [162]. Possible implications for CRC prognosis are shown by exosomal CPNE3. Patients diagnosed with CRC who had lower exosomal CPNE3 levels exhibited significantly improved DFS [hazard ratio (HR): 2.9; 95% confidence interval (CI): 1.3–6.4; *p* = 0.009] and OS (HR, 3.4; 95% CI: 1.2–9.9; *p* = 0.026) in comparison to patients with greater exosomal CPNE3 levels [66]. Numerous research investigations have demonstrated that small noncoding RNAs, including microRNAs contained in exosomes, can be found in serum and plasma in a stable form and may serve as potential biomarkers in a variety of malignancies, including CRC. Exosomal miRNAs in circulation could serve as biomarkers for CRC [163].

Exosomal miR‐431‐5p serves as a prognostic biomarker in CRC and low miR‐431‐5p expression was related to TNM stage, LNM, and differentiation degree. Patients with low miR‐431‐5p expression had a shorter OS [164]. Following surgical resections of CRC, patients with low expression of exosomal miR‐150 are predicted to have a poor prognosis. The OS time was considerably lower in patients with low expression of exosomal miR‐150 (*p* = 0.002). According to multivariate analysis, liver metastasis (*p* < 0.001) and low expression of exosomal miR‐150 (*p* = 0.035) were independent predictors of OS [165]. Poor prognosis in CRC is predicted by reduced serum exosomal miR‐874 expression. Low serum exosomal miR‐874 expression was linked to poor differentiation, advanced TNM stage, positive LNM, and positive distant metastasis [166]. Among CRC patients with liver metastasis, serum exosomal miR‐122 is a novel potential biomarker for prognosis. The results of univariate and multivariate logistic regression indicated that serum exosomal miR‐122 was a reliable predictor of CRC patients' outcomes [167]. Serum exosomal novel lncRNA NAMPT‐AS was identified as a potential biomarker for prognosis in patients with CRC. Compared to control subjects, CRC patients had considerably higher serum exosomal NAMPT‐AS levels, which also showed a strong positive correlation with serum exosomal NAMPT mRNA and circulating NAMPT protein [168]. Exosomal lncRNA HOTTIP is present in low amounts, and it acts as a prognostic indicator for CRC. The results of multivariate analysis indicate that HOTTIP is a reliable predictor of OS (HR: 4.5, CI: 1.69–11.98, *p* = 0.0027) [169].

### 1.16 Cervix Or Ovarian Cancer

Ovarian cancer is notorious for its resistance to chemotherapy, which unfortunately leads to a generally unfavorable prognosis. The 5‐year survival rate for OC is approximately 47%. However, there is a glimmer of hope, as early detection can significantly improve the chances of survival, with a relative survival rate of 93% [170]. Exosome‐related genes have the ability to predict the prognosis of OC patients. This is done by calculating the exosome‐related gene risk score based on the expression levels of certain immune checkpoint inhibitor targets. These targets include CD274 (PD‐L1), PDCD1 (PD‐1), PDCD1LG2, CTLA4, HAVCR2, and IDO1 [171]. The presence of plasma exosome‐derived fragile site‐associated tumor suppressor (FATS) has been found to be a reliable predictor of 5‐year survival in patients with OC. It has been observed that individuals with low levels of FATS tend to have a lower OC rate of 66% [172]. Serum exosomal miR‐484 is a reliable and non‐invasive indicator that can be utilized to predict the prognosis of OC. There is a strong correlation between lower blood exosomal miR‐484 expression and shorter OS, PFS, and aggressive clinical characteristics. The low serum exosomal miR‐484 level was confirmed through multivariate analysis, providing independent evidence [173].

### 1.17 Pancreatic Cancer

An aggressive and deadly cancer with a pitiful 5‐year survival rate is PDAC [174]. To precisely diagnose, categorize, and forecast the biological behavior of this tumor, there are no consistently dependable biomarkers or imaging modalities [175]. Recent research indicates that exosomes containing particular surface indicators or contents may be able to distinguish between PDAC patients and healthy persons [174]. When compared to the control group, the cancer group had a significantly higher serum level of Exo‐EphA2. The multivariate analysis revealed that high expression of Exo‐EphA2 in cancer was associated with a shorter OS and was a significant negative prognostic factor (HR = 1.04, 95% CI: 1.00–1.09, *p* < 0.001) [176]. Serum exosomal miRNA‐1226‐3p may be used as a biomarker to forecast PDAC tumor invasion or metastases. Serum exosomal miRNA‐1226‐3p expression was expressed at a lower level in PDACs than in benign pancreatic neoplasms (*p* = 0.025). miR‐1226‐3p performed satisfactorily in PDAC prediction with AUC = 0.74 [177]. Potential biomarkers for identifying patients at high risk of recurrence and poor survival in resected PDAC patients include exosome‐encapsulated miR‐4525, miR‐451a, and miR‐21 in portal vein blood. The results of a Cox regression analysis indicated that the levels of miR‐4525, miR‐451a, and miR‐21 in blood were independent predictors of both OS and DFS [178]. Exosomal miR‐200 as a predictor for liquid biopsy outcomes in PDAC. Shorter OS was found to be correlated with high expression of miR‐200c in total serum exosomes (*p* = 0.038) and miR‐200b in serum exosomes that were EpCAM‐positive (*p* = 0.032), while EpCAM exosomal miR‐200b was also associated with shorter OS in the subgroup of patients treated with curative intent (*p* = 0.013) [179]. There is prognostic significance of circulating exosomal proteins c‐Met and PD‐L1 for pancreatic cancer. Patients with c‐Met positivity exhibited a notably reduced postoperative survival duration (9.5 months vs. 21.7 months, *p* < 0.001). Additionally, patients with PDAC who tested positive for PD‐L1 had a much lower postoperative survival period (7.8 months vs. 17.2 months, *p* = 0.043) [83].

The quality of life and estimation of clinical outcome of therapeutic intervention of patients with cancers were assessed by analyzing the prognosis of the disease. Thus, the implication of effective and specific prognostic biomarkers, especially non‐invasive blood‐based markers, could aid the better management of cancer patients. They also can assist a medical oncologist in selecting more effective therapeutic regimen. Therefore, exosomal‐based prognostication could play vital roles in improving management of patients in point‐of‐care settings. However, further researches by incorporating larger cohorts are imperative for their validated prognostic applications in clinics.

## 2 Conclusions and Future Perspective

Cancer is a vast category of disorders that can originate in practically any organ or tissue when aberrant cells develop uncontrollably, invade neighboring organs, and spread through transferring the substances that are responsible for new tumor formation. Exosomes are crucial in the development of cancer since it has been considered active transporters and capable of transporting the contents of any type of cell. Exosomes produced by tumors aid in the development of the pre‐metastatic niche, angiogenesis, invasion, and therapeutic resistance of the tumor cells. Exosomes can be used as promising biomarkers for cancer diagnosis and prognosis because they closely resemble parent cells, are easy to collect from bodily fluids, and are stable in circulation. As we get a better grasp of exosome biology, exosome research is growing, and the corresponding diagnostic and prognostic approaches are also improving. Thus, reliable exosomal biomarkers in large samples are needed for early cancer detection and prognosis. In this review, we attempted to elucidate the types of exosomal components that serve as cancer biomarkers, the use of exosomal components in cancer diagnosis, based on which the disease can be detected, and the function of exosomal cargos in cancer prognosis, which can provide information about the disease's prognosis and predictions about its course. The potential use of exosomes in the future as biomarkers for the early diagnosis of cancer and the determination of a patient's prognosis will undoubtedly be a new weapon in the ongoing battle against cancer.

Although in the last decades the development of EV analysis impressively improved, their roles, mechanisms, and clinical implications in cancer are in the early stage. Therefore, there are still may issues, including time and quantity, mode of exosome collection, isolation, identification, and preservation methods, etc., that need to be resolved for their effective applications in clinical settings. Most importantly, techniques and platform for effective isolation and purification need to be developed and validated for clinical utility. Currently, the techniques that balance the vesicles' integrity, yield, and purity are necessary for effective and dependable extraction of exosomes. The gold standard for isolating exosomes, ultracentrifugation, has a high yield, is somewhat inexpensive, and has the following drawbacks: it is labor‐intensive and time‐consuming, may co‐isolate impurities like proteins and other vesicles, and requires specialized centrifuges. Size exclusion chromatography (SEC) preserves exosome integrity and bioactivity and has minimal protein contamination; however, it has moderate throughput and requires column preparation, which limit its applications. The process of ultrafiltration compared to SEC is quick and scalable, requires no high‐speed centrifugation, but has less purity. Though precipitation is easy to use and scalable, it has low purity (protein and lipoprotein co‐precipitation). An innovative technique for separating exosomes is microfluidic nanoparticle separation, which is a significant development, since it can solve a number of problems with traditional techniques, and it offers a high sensitivity and integrates isolation and analysis with a minimal sample size. However, more investigation is required to determine the most effective technique for isolating exosomes from cancer patients. It is also challenging to identify exosomes in cancer patients' biofluids, which calls for techniques that are effective and sensitive to their molecular and physical properties. These methods ought to be able to differentiate exosomes unique to cancer in order to aid in the development of diagnostic and treatment approaches.

In addition, in‐depth researches on the exact mechanism of their biogenesis, roles in cancer initiation, progression, cargo contents, and functions could provide critical insight for their potential applications. Thus, further studies and clinical trials are imperative to support the applications of exosomes in point‐of‐care settings. Integrated intensive innovative engineering techniques and basic researches could overcome these limitations. Developing efficient, robust, reliable isolation and characterization methods are urgent to further advancement of the field in translation in the future for better management of patients with cancers.

## Author Contributions

**Zinnat Ara Moni:** data curation (equal), resources (lead), writing – original draft (lead). **Zahid Hasan:** data curation (equal), resources (equal), writing – original draft (equal). **Md. Shaheen Alam:** data curation (equal), formal analysis (lead), investigation (equal). **Nitai Roy:** supervision (equal), writing – review and editing (supporting). **Farhadul Islam:** conceptualization (lead), supervision (lead), writing – review and editing (lead).

## Conflicts of Interest

The authors declare no conflicts of interest.

## Data Availability

All the information presented is referred in the manuscript.
